# Dissociate triggering of conjunctive and disjunctive eye movements

**DOI:** 10.1038/s41598-025-12031-5

**Published:** 2025-08-11

**Authors:** Baptiste Caziot, Frank Bremmer

**Affiliations:** 1 Applied Physics and Neurophysics, Philipps-Universität Marburg, Karl-von-Frisch Straße 8a, 35043 Marburg, Germany; 2 Center for Mind, Brain and Behavior, Universities of Marburg, Giessen and Darmstadt, Hans-Meerwein-Straße 6, 35032 Marburg, Germany

**Keywords:** Vergence, Saccades, Eye movements, Binocular vision, Human behaviour, Visual system

## Abstract

In natural behavior, our eyes must coordinate two types of movements when looking between points in space: conjunctive movements (where both eyes move together) and disjunctive movements (where the eyes move in opposite direction to change their convergence angle). Here we investigate how the initiation of these 2 different types of eye-movements is coordinated. We used the Size-Latency effect to modulate saccadic latencies. To elicit combined saccadic and vergence eye-movements, we displayed large ring targets at different vertical offsets and disparities relative to fixation. This allowed us to easily dissociate version and vergence eye-movements. We found that saccadic latencies were strongly modulated by the eccentricity of the targets as well as their hemifield, but not by the disparity of the targets. The opposite was true for vergence: vergence latencies were modulated by the disparity sign and amplitude of the targets, but not by their eccentricity or hemifield. We found a complete lack of correlation between saccadic and vergence latencies, both across and within conditions. Finally, we found that distributions of vergence latencies have a markedly reduced skewness as compared to distributions of saccadic latencies, a hallmark of evidence accumulation. Overall, our results demonstrate that the initiation mechanisms for these two types of eye-movements operate independently.

**Supplementary Information:**

The online version contains supplementary material available at 10.1038/s41598-025-12031-5.

## Introduction

Two points in a visual scene most likely differ in both (horizontal and/or vertical) direction and distance. When fixating at these two separate locations, the eyes of an observer have different *version* (average orientation of the 2 eyes) and *vergence* (convergence angle between the 2 eyes). Consequently, to move from one point to the other, the 2 eyes have to move by a different amount. Hering’s law of equal innervation states that “the musculature of both eyes reacts simultaneously to one and the same impulse of will”^1^or in other words that the eyes always move by equal amounts, if not always in the same direction. So, moving the eyes between 2 points in space require coordinating a *conjunctive* eye-movement (where the eyes move in the same direction, typically through a saccade), with a *disjunctive* eye-movement (where the eyes move in opposite direction to change their convergence angle). During natural behavior, conjunctive eye-movements are almost always co-occurring with disjunctive eye-movements^2^; except in special cases where points have the same distance but different direction, or the same direction but different distances, which represents the overwhelming majority of experimental studies. It remains unclear how these two radically different types of eye-movements are coordinated. The purpose of this study was to investigate a single aspect of their coordination: their initiation.

Various effects are known to modulate saccadic latencies such as contrast of a saccade target (e.g^3^). Phenomena based on supra-threshold stimuli typically cause increases on a more limited scale. Inhibition of return, for instance, is typically associated with delays in saccade execution between 10 and 40 ms (reviewed in^4^). Remote distractors that onset around the time of the target lead to a latency increase of around 20–30 ms^5,6^. This increase just exceeds 50 ms when the distractor is placed at fixation^7^. A manipulation that has been shown to cause more considerable fluctuations in latencies is the gap effect. There the removal of the fixation marker 200 ms prior to target onset decreases latencies considerably. However, while the initial findings surrounding the gap effect showed latency decreases up to 100 ms^8,9^ later findings reported much smaller effects^10,11^. It is also likely that aside from the benefit of releasing inhibition at fixation, the offset serves as a warning signal for the upcoming stimulus^8,12–14^. Indeed, similar effects are observed for actions that do not involve moving the eyes, such as swallowing^15^. Finally, other factors, such as target location predictability have been shown to also contribute to the gap effect^16^. Hence, the great reduction in latencies appears to be a compound of multiple effects.

Two studies have looked specifically at the initiation of conjunctive and disjunctive eye-movements using a gap paradigm^17,18^. These studies both found substantial correlations between the initiation latency of version and vergence eye-movements. While one study concluded that the initiation of version and vergence eye-movements are independent despite their correlation^17^, the other study concluded that their initiation is coordinated to some extent^18^. Therefore, whether the initiation of version and vergence eye-movements are, or are not, coordinated remains an outstanding question. Furthermore, as stated above, there are suspicions that part of the gap effect is a compound of multiple effects, complexifying interpretation of these studies.

Here we used the Size-Latency effect^19–22^, an effect known to strongly modulate saccadic latencies: saccades towards large targets are strongly delayed, by sometimes over 100 ms, as compared to saccades towards small targets. Furthermore, this effect is better explained by the relative size of a target as compared to its eccentricity, reflecting magnification of the neural representation of the visual field (typically termed cortical magnification) observed in the cortex or in Superior Colliculus. Here, we used this effect to artificially delay saccades during joint saccade-vergence eye-movements. Our results show that vergence initiation latencies remained unchanged even when saccadic latencies were strongly increased; that saccadic latencies were unchanged when vergence latencies changed; and that there was complete lack of correlation between saccadic and vergence latencies. This demonstrates that the initiation mechanisms for these two types of eye-movements operate independently.

## Results

**Fig. 1:**
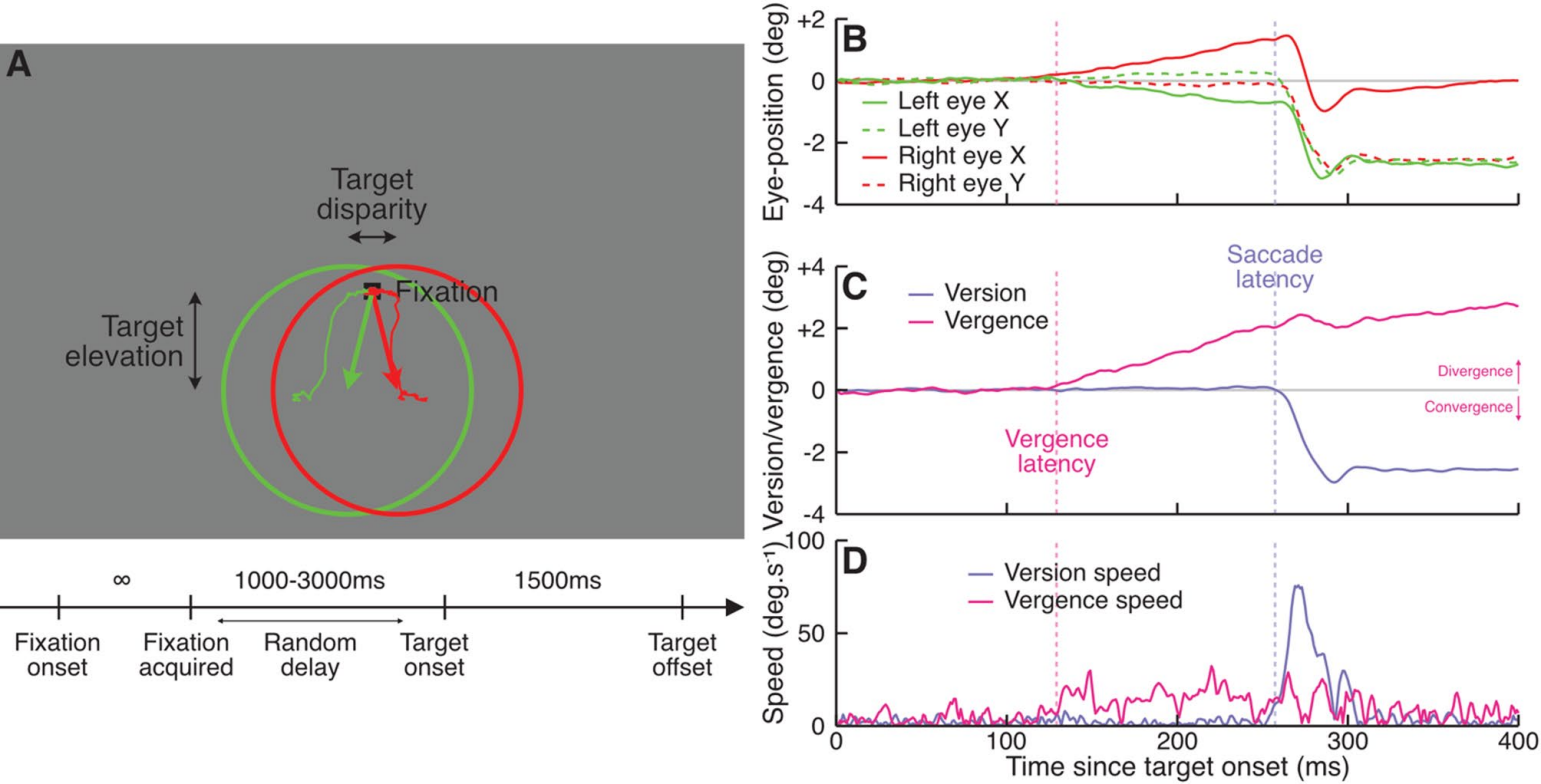
(**A**) Depiction of the stimulus. Observers fixated a small fixation mark at the center of the display. After a random delay, the fixation mark was replaced by a large ring target with varying vertical offsets (elevation) and binocular disparity relative to fixation. Observers were instructed to fixate the center of the ring. The arrows show how much the eye should move from fixation to the center of the ring. The thin lines show actual eye traces in a trial with similar target elevation and disparity as depicted (same trial as in **B**–**D**). (**B**) Example of eye-position signals during a trial relative to target onset (abscissa). Here the eyes started deviating horizontally in opposite directions (red and green solid lines) approximately 125 ms after target onset. Approximately 260 ms after target onset, both eyes jumped vertically in the same direction (red and green vertical dotted lines) by about 2 degrees. (**C**) Version (purple) and vergence change (pink) as a function of time relative to target onset. The vergence angle corresponds to the difference between solid lines in (**B**). The version angle corresponds to the mean of the dashed lines in (**B**). (**D**) Version speed (purple) and vergence speed (pink) as a function of time relative to target onset.

Stimuli were presented through an anaglyph stereoscopic display (red/green filters)^23 ^allowing us to display stimuli independently to each eye: red points appeared black when seen through the green filter, but were nearly invisible when seen through the red filter; and conversely for green points. By introducing an offset (binocular disparity) between the position of a point in the left and right eyes, we simulated stimuli nearer than the display (crossed disparity), eliciting a converging eye-movement; farther than the display (uncrossed disparity), eliciting a diverging eye-movement; or at the same distance as the display (0-disparity), eliciting no vergence response.

Observers were instructed to fixate a small fixation mark (30 arcmin) at the center of the display (see Fig. 1A). After a random delay between 1000 and 3000 ms, the fixation mark was replaced by a large ring target (12 deg diameter). The center of the ring had one of 7 vertical offsets (or elevation) of 0, ±2, ±4 or ±6 deg. The ring also had one of 5 binocular disparities of 0, ±1 or ±2 deg relative to fixation, independently from eccentricity; with the exclusion of the condition with 0-offset and 0-disparity which would not have elicited any eye-movement. Observers were instructed to try to look at the center of the ring when it appeared.

Figure 1B shows an example of eye-position signals for the same trial as plotted in Fig. 1A, as a function of time relative to target onset. The horizontal positions of the eyes (red and green solid lines) started deviating in opposite directions 125 ms after target onset. The vertical positions of the eyes (red and green dashed lines) remained stable until 260 ms where both eyes abruptly moved downward, a saccadic eye-movement. Both eyes also moved horizontally by a similar amount, because the observer did not make a perfectly vertical saccade. Figure 1C shows vertical *version* (mean vertical eye-position of both eyes, purple line) and *vergence* (difference between the horizontal position of the eyes, pink line) for the same trial. Vergence started deviating from baseline when the eyes started moving horizontally in opposite direction, and slowly increased for the next 200 ms until reaching approximately  2 deg. Version remained near baseline until 260 ms, at which point vertical version changed from near 0 to near - 2 deg in the space of approximately 50 ms. Vergence remained largely unchanged despite the fairly large horizontal component of the saccadic eye-movement. Finally, Fig. 1D plots version and vergence speed, the derivative of signals plotted in Fig. 1C. While saccade onset (purple dotted line) is easy to detect from the large change in version speed, it is more difficult to detect vergence onset (pink dotted line) due to the lower signal-to-noise ratio of vergence speed. Instead, we adopted a regression-based approach to detect vergence eye-movements onset^24–26^. Evaluation of this approach through simulations (see Figure S1) indicates that it allowed detecting vergence onsets accurately, with only a small (4.5 ms) underestimation of the true values.

**Fig. 2:**
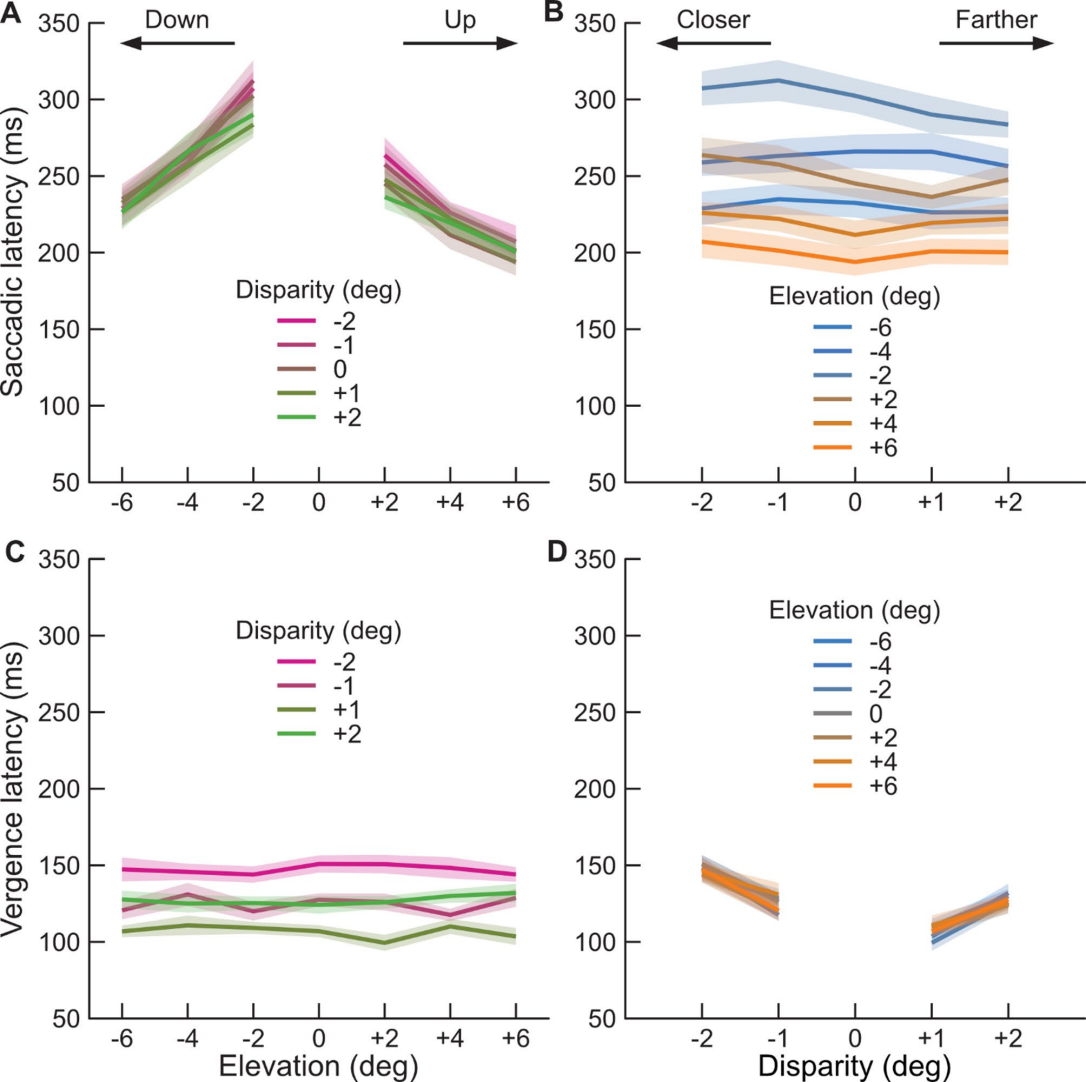
(**A**) Mean *saccadic* latencies across observers (thick lines) and standard error (shaded area) as a function of target elevation (abscissa) and target disparity (colors). (**B**) Same as A plotted as a function of disparity (abscissa) and target elevation (color). (**C**) Mean *vergence* latencies across observers (thick lines) and standard error (shaded area) as a function of target elevation (abscissa) and target disparity (colors). (**D**) Same as (**C**) plotted as a function of disparity (abscissa) and target elevation (color).

### Saccadic and vergence latencies

Figure 2A plots the mean saccadic latencies across observers as a function of target elevation. The first noticeable effect is the asymmetry between saccades towards targets in the upper and lower hemifields. The mean saccadic latency across observers, disparities and eccentricity was 264 (± 10 SE) ms for downward saccades vs. 225 (± 9 SE) ms for upward saccades, a difference of 39 ms.

The second noticeable effect is the drop in saccadic latencies with eccentricity. Because the target size was fixed to 12 deg, an increase in eccentricity corresponds to a reduction in the target Distance-to-Size Ratio (DSR), the so-called Size-Latency effect. As expected, saccadic latencies decreased as the eccentricity increased. The mean latency across observers, disparities and hemifield at 2 deg eccentricity was 275 (± 8 SE) ms vs. only 215 (± 9 SE) ms at an eccentricity of 6 deg, a difference of 60 ms.

The last noticeable effect is the near perfect overlap between curves for different binocular disparities: the disparity of the target did not appear to produce any meaningful modulation of saccadic latencies. This lack of effect is clearest in Fig. 2B which plots saccadic latencies as a function of disparity. Negative values correspond to crossed disparities (target closer than fixation) and positive values to uncrossed disparities (target farther than fixation). Lines are mostly flat and no obvious trend is present.

To assess significance of these effects, and their possible interactions, we performed a four-way ANOVA with factors *hemifield* (upper/lower), *eccentricity* (2, 4, 6 deg), *disparity sign* (crossed/uncrossed) and *disparity value* (1, 2 deg). As is clear in Fig. 2A, both *hemifield* and *eccentricity* were significant: respectively F(26.2,1) = 74.5, *p* < 0.001 and F(45.8,2) = 65.0, *p* < 0.001. The interaction between these 2 factors was not significant: F(0.1,2) = 0.1, *p* = 0.89. The other 2 main factors, related to the disparity of the target, were not significant, and neither were all possible remaining interactions (see Table S1).

We confirmed the effect of hemifield and eccentricity in ad-hoc t-tests on the mean saccadic latencies across observers. The mean saccadic latency was significantly longer for an eccentricity of -2/+2 degrees as compared to an eccentricity of -6/+6 degrees (t-tests, respectively t(10) = 10.5, *p* < 0.001 and t(10) = 13.3, *p* < 0.001). Similarly, the mean saccadic latency was significantly higher for eccentricities of + 2/+6 (lower hemifield), as compared to eccentricities of -2/-6 (t-tests, respectively t(10) = 4.1, *p* = 0.002 and t(10) = 4.9, *p* = 0.001).

In summary saccadic latencies were modulated by the hemifield (upper vs. lower) where the target was presented, as well as by the eccentricity of the target. These effects appeared to be independent, and saccadic latencies appeared unmodulated by the disparity of the target.

Figure 2C plots mean vergence latencies across observers as a function of target eccentricity. Vergence latencies are overall dramatically shorter than saccadic latencies. The average median saccadic latency across conditions and observers was 244 (± 8 SE) ms, and the average median vergence latency only 126 (± 3 SE) ms. This near doubling of latency was significant in a pairwise t-test (t(10) = 15.8, *p* < 0.001).

No clear trend in the vergence latencies as a function of target eccentricity is visible. However, vergence latencies seem clearly modulated by the target disparity. This effect is clearer in Fig. 2D, which plots mean vergence latencies across observers as a function of target disparity. Crossed disparities (negative values) require convergence of the eyes and uncrossed disparities (positive values) require divergence of the eyes. Here vergence latencies seem to increase slightly with the disparity of the target (and therefore the amplitude of the vergence eye-movement) with mean latencies of 116 (± 2 SE) ms for 1 deg of disparity and 137 (± 4 SE) ms for 2 deg of disparity. Similarly, vergence latencies are lower for targets with uncrossed disparities (divergence) than targets with crossed disparities (convergence) with mean vergence latencies of respectively 117 (± 3 SE) ms and 136 (± 4 SE) ms.

We assessed significance in a similar four-way ANOVA. As suggested by the data plots, the factors *disparity sign* and *disparity valu*e were significant (respectively F(93.0,1) = 57.1, *p* < 0.001 and F(147.2,1) = 90.3, *p* < 0.001). The factors hemifield and eccentricity were not significant, neither were any possible interactions between variables (see Table S2).

We confirmed these effects in ad-hoc t-tests on the mean vergence latencies across observers. The mean vergence latency was significantly longer for a disparity of -2/+2 degrees as compared to a disparity of -1/+1 degrees (t-tests, respectively t(10) = 7.2, *p* < 0.001 and t(10) = 8.4, *p* < 0.001). Similarly, the mean vergence latency was significantly lower for disparities of + 1/+2 deg (uncrossed disparities/divergence), as compared to disparities of -1/-2 (t-tests, respectively t(10) = 4.4, *p* = 0.001 and t(10) = 3.8, *p* = 0.004).

In summary, vergence latencies were modulated by the disparity of the targets, both by the disparity amplitude and sign, and these effects appeared independent. But the hemifield and eccentricity of the targets did not appear to modulate vergence latencies.

**Fig. 3:**
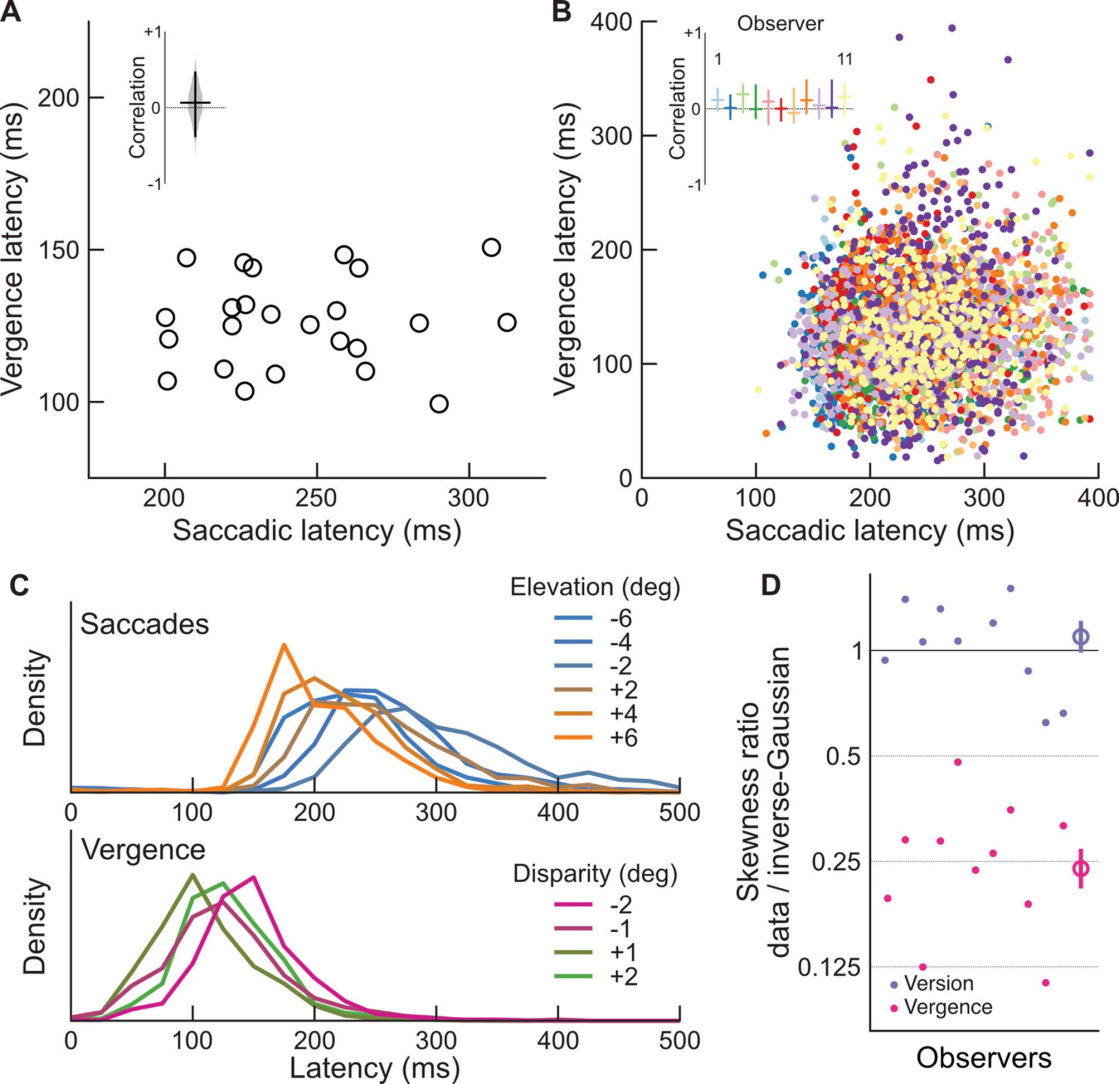
(**A**) Mean vergence latency (ordinates) across observer for each stimulus condition as a function of saccadic latency (abscissa). The inset shows the median correlation between vergence and saccadic latencies (horizontal line), the distribution of correlation factor when resampling the dataset (violin plot), and its 95% confidence interval (vertical line). (**B**) Vergence latency (ordinates) as a function of saccadic latency (abscissa) for each trial and observer separately. The inset shows the median Spearmann’s rank correlation coefficient and its 95% confidence interval for each observer separately. (**C**) Top: mean distributions of saccadic latencies across observers as a function of target elevation (colors). Bottom: mean distributions of vergence latencies across observers as a function of target disparity (colors). (**D**) Ratio of the skewness of the saccadic and vergence distributions (respectively purple and pink) over the skewness of an inverse-Gaussian distribution of identical mean and variance (ordinates) for each observer separately (abscissa), plotted on a log-scale. Circles on the right show mean skewness ratio and vertical lines the standard error. Here a ratio of 1 indicates that the actual distribution is as heavily skewed as an inverse-Gaussian, and a ratio lower than 1 indicates that the distribution is more symmetrical than an inverse-Gaussian.

### Relationship between saccadic and vergence latencies

We then focused on the relationship between saccadic and vergence latencies. Figure 3A plots mean vergence latencies as a function of mean saccadic latencies across observers, for conditions that elicited both saccadic and vergence eye-movements (where both eccentricity and disparities were non-zero). The correlation between saccadic and vergence latencies was 0.07, which is extremely weak. We assessed significance by resampling the mean saccadic and vergence latencies across conditions and computing the correlation factor for each sample. The 95% confidence interval of the correlation factor in this analysis was [-0.39, + 0.48], which includes 0 (see inset in Fig. 3A).

Figure 3B plots vergence latencies as a function of saccadic latencies for each trial and observer separately. We assessed trial-to-trial correlations within conditions for each observer separately using Spearmann’s rank correlation. This correlation coefficient was on average 0.06. We resampled data for each subject separately in order to compute the within-subject 95% confidence interval of this correlation factor, and found that it was narrowly centered on 0 for every observer (see inset in Fig. 3B). The mean lower and upper bounds of these confidence intervals was [-0.13, 0.29].

We also analyzed the shape of the distributions of saccadic and vergence latencies. Distributions of saccadic latencies typically exhibit a right heavy-tail and are well approximated by an inverse-Gaussian distribution, the continuous limit of Wald’s sequential probability ratio test^27,28^. This long-noted observation is largely understood as demonstrating that saccadic initiation is a *deliberative* process in which observers accumulate a noisy signal toward a fixed level of confidence about the presence of a target^29,30^. Figure 3C plots the distributions of saccadic latencies as a function of target elevation on top, and the distributions of vergence latencies as a function of target disparity on the bottom. The distributions of vergence latency do not seem to exhibit as strong a heavy-tail as the distributions of saccadic latency. To assess this effect, we computed the mean skewness of these distribution across conditions (see Methods). The skewness was on average 0.56 (± 0.2 SE) for saccadic distributions, and 0.24 (± 0.2 SE) for vergence distributions. Both these values are significantly higher than 0 in a t-test (t(10) = 11.0, *p* < 0.001 and t(10) = 7.0, *p* < 0.001), indicating some degree of right-skewness for both. We then compared this skewness to the skewness of an inverse-Gaussian of identical mean and variance by computing the log-ratio of these metrics (see Fig. 3D). While we found, in agreement with the rest of the field, that distributions of saccadic latencies are well approximated by an inverse-Gaussian distribution, as demonstrated by a skewness log-ratio not significantly different from 0 (t(10) = 1.2, *p* = 0.27); the skewness of vergence latency distributions were significantly lower (t(10) = 11.0, *p* < 0.001), only about 24%, than the skewness expected from an inverse-Gaussian distribution. This indicates that while saccadic latencies are well approximated by a heavy-tail distribution, such as an inverse-Gaussian distribution, vergence latency distributions are more symmetrical, closer to a normal distribution, and therefore are unlikely to be well described by a stochastic accumulator decision model.

**Fig. 4:**
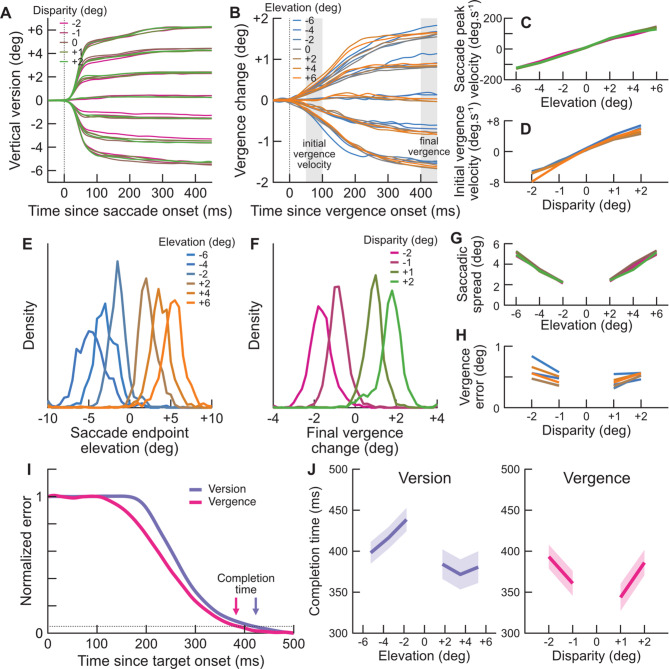
(**A**) Mean vertical version across observers (ordinates) as a function of time since saccade onset (abscissa) for each target elevation and disparity separately. The vertical dashed line represents eye-movement onset. Target elevations are unlabeled for clarity, but are obvious from the grouping of the eye traces. For conditions that did not elicit saccadic eye-movements (target centered at fixation), we aligned eye traces to the median saccadic latency across conditions. (**B**) Mean vergence change across observers (ordinates) as a function of time since vergence eye-movement onset (abscissa) for each target distance and disparity separately. The vertical dashed line represents eye-movement onset. Target disparities are unlabeled for clarity, but are obvious from the grouping of the eye traces. The 2 shaded areas represent the time windows used for computing vergence velocity (50–100 ms) and final vergence change (400–450 ms). For conditions that did not elicit vergence eye-movements (target with 0-disparity), we aligned eye-traces to the median vergence latency across conditions. (**C**) Mean saccadic peak velocity (ordinates) as a function of target elevation (abscissa) and disparity (colors). (**D**) Mean vergence velocity within a 50–100 ms time window after vergence eye-movement onset (ordinates) as a function of target disparity (abscissa) and elevation (colors). (**E**) Distributions of the saccadic endpoints elevation as a function of target elevation (colors). (**F**) Distribution of change in vergence eye-posture within a 400–450 ms time window after vergence onset as a function of target disparity (colors). (**G**) Mean spread of saccadic endpoints across observers (standard deviation of the distribution) as a function of target elevation (abscissa) and disparity (colors). Here the legend has been omitted for clarity and colors correspond to the legend of (**F**). (**H**) Mean vergence error across observers (absolute difference between vergence change and target disparity) within a 400–450 ms time window after vergence onset as a function of target disparity (abscissa) and elevation (colors). Here the legend has been omitted for clarity and colors correspond to the legend of (**E**). (**I**) Mean normalized elevation error (purple) and normalized vergence error (pink) across observers and conditions as a function of time since target onset (abscissa). Arrows represent completion time estimated as the time where 95% of the initial error has been corrected. (**J**) Left: mean version error-correction completion time since target onset (ordinates) as a function of target elevation (abscissa). Shaded areas are standard errors. Right: mean vergence error-correction completion time since target onset (ordinates) as a function of target disparity (abscissa). Shaded areas are standard errors.

### Dynamics and accuracy of saccadic and vergence eye-movements

Finally, we looked at the dynamics and accuracy of eye-movements. Figure 4A plots the mean vertical version across observers as a function of time relative to saccade onset. As expected, version changes abruptly within 50ms from the central fixation location to near the center of the target. Eye-position keeps approaching the targets center after the initial saccadic eye-movements because observers often performed a second corrective saccade.

Figure 4B plots the mean vergence change across observers as a function of time relative to vergence onset. Here a much slower change in eye-position can be observed, as compared with saccades. Vergence changes in the direction of the disparity of the target over the course of roughly 400ms, following approximately an exponential ramp typical of closed-loop systems. A small difference can be observed as a function of target elevation.

Figure 4C plots the mean saccadic peak velocity across observers as a function of target elevation and disparity. The elevation of the target clearly modulates peak velocity, in broad agreement with the main sequence. Saccade peak velocities appear slightly higher for downward saccades than upward saccades. Finally, disparity did not appear to modulate saccadic velocities. We confirmed these effects by fitting a linear model to the data with target elevation and disparity as regressors. The elevation factor significantly modulated peak velocities (β = 23.1, SE = 0.3, *p* < 0.001), but not disparity (β = 0.0, SE = 1.0, *p* = 0.96). The intercept term, corresponding to an asymmetry between upward and downward peak velocities, was also significant (β=-10.0, SE = 1.3, *p* < 0.001).

Figure 4D plots mean initial vergence velocity across observers, computed within a 50–100 ms window after vergence onset, as a function of target disparity and elevation. Vergence velocity relates almost linearly with target disparity. Moreover, converging eye-movements (blue) appears slightly faster for targets in the lower hemifield (negative elevation), and diverging eye-movements (orange) appear slightly faster for targets in the upper hemifield (positive elevations). We fitted again a linear model and this model did not have a significant intercept (β = 0.0, SE = 0.1, *p* = 0.78), indicating approximately equal vergence velocities for converging and diverging eye-movements. However, both the disparity and eccentricity factors significantly modulated vergence velocities (respectively β = 3.0, SE = 0.08, *p* < 0.001 and β = 0.08, SE = 0.03, *p* = 0.009). Note that this effect is not caused by saccadic eye-movements, since we computed vergence velocities in a window that generally precedes saccade onset. Indeed, we repeated the same analysis while computing vergence velocities in a window of -50 to 0 ms *prior* saccade onset and found identical results. However, a linear model fitted on vergence velocities computed in a window of 0 to 50ms *after* saccade onset (during the saccade) did not have a significant eccentricity factor. Finally, in the vast majority of trials saccade onset starts long after vergence onset (between 70 and 170 ms depending on the target elevation). However, when vergence and saccade onsets are in close proximity we observed a clear enhancement in the initial vergence velocity (see Figure S2).

We then looked at the accuracy of saccadic and vergence eye-movements. Figure 4E plots the distribution of saccadic endpoints (the eye-position at the end of the first saccade after target onset), as a function of target elevation (colors). The spread of saccadic endpoints tends to increase slightly with the eccentricity of the target, as can be seen in Fig. 4G which plots the mean spread of saccadic endpoints as a function of target elevation and disparity. We conducted similar four-way ANOVAs as for eye-movement initiation times, and found that only the factor eccentricity significantly modulated saccadic spread (F(341.5,2) = 141.8, *p* < 0.0001, see Table S3), confirming that the effect of eccentricity on saccadic latency is not a confound of a higher spread due to the target disparity.

Figure 4F plots the distributions of final change in vergence within a time window of 400–450 ms after vergence onset as a function of target disparity. These distributions seem slightly wider for targets with higher disparity. Figure 4H plots the mean vergence error (absolute difference between final vergence change and target disparity) as a function of target disparity and elevation. A four-way ANOVAs confirmed the factors *disparity value* and *eccentricity* both significantly modulated vergence errors (respectively F(1.2, 1) = 8.38, *p* = 0.004 and F(1.3, 2) = 4.32, *p* = 0.01). None of the other factors or interactions were significant (see Table S4).

As a final analysis, we looked at the overall dynamics of error-correction. Eye-position error, corresponding to the Euclidian distance between the current eye-position and the target center, is highest at target onset and tends to decrease over the course of a trial. For each condition and observer, we computed a normalized error (see Methods) by subtracting the minimum mean error over the course of a trial for a given observer and condition, and dividing by the maximum mean error. This normalized error is close to 1 at target onset, and reaches 0 near the end of the trial, when the eyes are closest to the target center. Figure 4I plots this mean normalized error across observers and conditions, for both version and vergence, as a function of time since target onset. The dynamics of version and vergence error correction parallel each other, and are in fact slightly preceded by vergence. We then computed an “error completion time”, measured as the first time where over 95% of the starting normalized error has been corrected. These mean completion times across observers can be seen in Fig. 4J on the left for version as a function of target elevation, on the right for vergence as a function of target disparity.

## Discussion

Here we investigated the initiation of combined saccadic and vergence eye-movements toward targets that varied in eccentricity and disparity. By varying the target eccentricity while maintaining its size constant, we expected to modulate saccadic latencies according to the Size-Latency effect^19,20^. We paid close attention to the relative initiation of vergence eye-movements as compared to saccadic eye-movements.

Regarding saccadic eye-movements, we found first a clear asymmetry between the upper and lower visual fields. This asymmetry has been previously documented for both saccadic and smooth-pursuit eye-movements^31–36^. This asymmetry in initiation latencies is mirrored by a stronger representation of the upper visual field in the Superior Colliculus^34,37,38^. An opposite asymmetry exists for visual perception during steady-gaze, where performance is typically better in the lower visual field, mirrored by a stronger representation of the lower hemifield in the cortex^38,39^. Consequently, the benefit of the upper visual field for saccadic initiation very likely originates in SC.

Secondly, we found, as expected, an effect of the target eccentricity on saccadic latencies, with smaller saccades requiring longer initiation latencies. We recently showed that this effect can also be accounted for, at least in part, by activity in the Superior Colliculus^27^. We found that large ring stimuli inhibit the visual responses of SC cells, which leads to weaker and delayed motor responses of the same cells. Consequently, this effect most likely also originates in the SC, and is likely related to other effects that have been suggested to be caused by lateral inhibition in the SC, such as the Remote Distractor Effect^40^. We found no interaction between this effect and the hemifield effect suggesting that they are independent.

Finally, we found no effect of the target disparity on saccadic latencies. Therefore, saccadic latencies seem to be purely a function of the target spatial (2D) location irrespective of the depth of the target. One interesting implication of this result is that the Size-Latency effect seems to be a function of the *retinal* size of the target, not its *perceived* size. Here we used large disparities that produced noticeable differences in perceived size between crossed and uncrossed disparities. In our stimulus, a target of fixed retinal size and 2 deg of uncrossed disparity (farther than fixation) would be, geometrically, nearly twice as large as a target of same retinal size with 2 deg of crossed disparity (closer than fixation). Even though size constancy is incomplete^41^and stimuli were clearly diplopic due to the large disparities, we would expect the difference in perceived size of the target to be large enough to produce meaningful modulations in saccadic latencies. The fact that they did not suggests that the signals involved in the Size-Latency effect are not involved in perception, in good agreement with the idea that this effect originates in the Superior Colliculus.

Regarding vergence eye-movements, we found first that they are initiated far earlier than saccadic eye-movements, a well-known result documented from the earliest studies on vergence eye-movements^42,43^. These earlier studies reported vergence latencies in the order of 160 ms. Here we found latencies in the order of 130 ms. This difference might be due to the relatively large targets we used. Even shorter vergence latencies, in the order of 80 ms, have been reported using very large scale stimuli^44,45^.

Secondly, we found an asymmetry between targets with crossed and uncrossed disparities (eliciting respectively converging and diverging eye-movements), with a slightly faster initiation of diverging eye-movements. Asymmetries in initiation latencies between converging and diverging eye-movements have been highly inconsistent in the past literature. Some studies report a shorter initiation latency for converging eye-movements^11^others for diverging eye-movements^18,46–48^and some found no difference^18^. These differences could be due to large inter-individual variations^17,46^or to differences between stimulus and apparatus. Most notably, most of these studies used combination of horizontal saccades and vergence eye-movements. In these circumstances, it is difficult to dissociate the onset of vergence from the inherent changes in horizontal alignment that occur naturally during horizontal saccades^49,50^. Here, we used vertical saccades to easily dissociate version from vergence. Furthermore, this asymmetry between crossed and uncrossed disparities was consistent across stimuli and observers.

Thirdly, we also found slightly shorter initiation latencies for smaller vergence eye-movements than larger vergence eye-movements. The reason for this difference is unclear, and goes in the opposite direction of the Size-Latency effect for saccades, where smaller eye-movements have longer initiation latencies. Furthermore, this effect is present for both crossed and uncrossed disparities, excluding the possibility that this effect is related to the perceived size of the stimulus. This effect was consistent across target elevation and independent from the effect of disparity sign. But it is possible that this effect is simply a byproduct of the regression-based approach we used to detect initiation latencies. More extensive measurements would be required to assess its veracity.

Finally, we found no effect of target hemifield or eccentricity on vergence latencies. This is in contrast with prior studies that found an effect of target sizes and eccentricity on the response gain of vergence eye-movements to oscillating stimuli^51,52^. This might reflect different spatial tuning for different components vergence eye-movements control^53^.

We then computed the correlation between saccadic and vergence initiation latencies, both across and within observers. In both cases their correlation coefficient was narrowly centered on 0 and reached significance in none of the observers. This is the main result of our study. While previous studies using the gap effect found substantial correlations between saccade and vergence initiation latencies^17,18^here we found a complete lack of correlation. This result suggests that changes in eye-movements initiation latency produced by the gap effect are likely caused, at least in part, by other mechanisms than the eye-movement decision process^12,13^and that the Size-Latency effect is better suited for producing modulations of saccadic latencies devoid of these confounds^20^.

Here we used vertical saccades for 2 reasons: firstly, it allowed easily dissociating horizontal vergence eye-movements from vertical saccadic eye-movements. Secondly, we expected reduced interactions between version and vergence signals, though vergence has been reported to be modulated by vertical saccades as well as horizontal ones^49,54–56^. One could ask whether our results remain true for joint vergence and horizontal saccades. If the triggering of horizontal saccades were not independent from vergence, as we have shown here for vertical saccades, then this result would imply that the triggering of the horizontal component of a saccade is itself at least partially independent from the triggering of the vertical component. Since this is contradictory with everything that is known about the physiology of saccadic eye-movements^57^we can safely conclude that our result would hold for horizontal as well as vertical saccades, or any combination thereof.

We also found that distributions of vergence latencies exhibited a markedly reduced heavy-tailedness as compared to distributions of saccadic latencies, an effect generally understood as reflecting evidence accumulation of a noisy signal within a saccadic decision-making process^29,30^. This result is important because even if vergence eye-movements are triggered independently from saccadic eye-movements, as we argue here, they could still rely on a similar mechanism. Manual response times, for instance, are also well described by evidence accumulator models (e.g^58^. for a review). Our results demonstrate that vergence initiation cannot be well described by such class of models, and consequently that the mechanism triggering vergence eye-movements is qualitatively different than the one for saccades. However, this result should be interpreted with one potential caveat: our regression-based approach for estimating vergence onset could potentially underestimate the distribution’s skewness. Our analyses (see Figure S3) suggest this methodological concern is unlikely to explain our findings. Instead, the reduced skewness we observed appears to reflect a genuine difference in vergence onset timing rather than measurement noise.

We then investigated the dynamics and accuracy of saccadic and vergence eye-movements across stimulus conditions. Many of the results reported here are already known. For instance, the increase of saccadic peak velocities and saccadic spread with eccentricity^59^ as well as the near linear relationship between target disparity and vergence velocity within this range of disparities^44,45,60^. What is notable is that the disparity of the targets never modulated saccadic eye-movements, neither their latency, their velocity, nor their accuracy. In contrast, we found that the eccentricity of the targets did modulate the dynamics of vergence eye-movements: converging eye-movements were faster for targets in the lower hemifield and diverging eye-movements for targets in the upper hemifield. This effect has previously been reported^2,61^but has been attributed to interactions between the saccadic and vergence systems. Our results suggest that this effect is due to the vergence system alone, although our study was not especially designed to investigate this effect. Similarly, we found that vergence error increased with target eccentricity. But note that here we did not take into account changes in vergence due to eccentricity alone (due to our planar display), and this effect might disappear under a more rigorous treatment of vergence eye-posture. Finally, we found that vergence velocity was enhanced by the occurrence of saccades (see Figure S2). This effect has sometimes been interpreted as a violation of Hering’s law. Instead, the clear lack of coordination between the initiation of saccadic and vergence eye-movements provides strong evidence that this enhancement is caused by downstream interactions between the version and vergence motor signals (see next section), as suggested by previous studies^54–56,62,63^.

Finally, we investigated the dynamics of saccades and vergence as error-correction systems. That is, as a system that tends to minimize errors between the position of the eyes and the position of the target. Despite the much lower speed of vergence eye-movements, which rarely exceeded 8 deg.sec^−1^ in our study (see Fig. 4C), as compared to saccadic peak velocities, which typically exceeded 100 deg.sec^−1^ (see Fig. 4D); we can see in Fig. 4I that *on average* vergence eye-movements reach their final position at a similar time, if not slightly prior, as compared to version eye-movements. This difference is due to both the shorter initiation delays of vergence eye-movements (Fig. 2), as well as the lower variability in their initiation time (Fig. 3C,D). This also means that the dynamics of error-correction in version varies widely from trial-to-trial. In some trials, the eyes reach the same elevation as the target in as shortly as 200ms. On some other trials, the eyes are still at fixation almost 400 ms after target onset. In contrast, the dynamics of vergence error changes fairly reliably across trials. Furthermore, the specific dynamics for either eye-movement change slightly depending on specific stimuli (Fig. 4J), and under different circumstances saccadic eye-movements would be completed faster than vergence. Nonetheless, if as we argue here saccadic and vergence eye-movements are separate systems that operate independently from each other, it seems that their respective dynamics are tailored in such a way that the eyes reach the target location *on average* at approximately the same time (~ 400 ms) for both vergence and saccadic eye-movements.

### Physiological considerations

Some interactions between version and vergence eye-movements are known to exist^49,55,56,64–67^ (and see Figure S2). These observations led some to reject Hering’s law of equal innervation and propose that the eyes are controlled monocularly^49,68,69^. Others have instead suggested that because both vergence and version eye-movements are controlled by the same muscles, they are triggered by the same motor neurons. This simple physiological constraint gives opportunity for version and vergence signals to interact^54,55,62,67^potentially explaining these interactions without rejecting Hering’s law. Our results strongly suggest that there is no coordination between saccades and vergence, since it is hard to see how these eye-movements could be triggered completely independently, while their programming is partially coordinated. We conclude, as others have before us^55,56,62,63^that Hering’s law remains valid and that saccade-vergence interactions are simply caused by the temporal co-occurrence of these 2 types of eye-movements.

Physiological correlates of vergence eye-movement prior to that stage remain unknown. Three areas have been identified as potentially involved: Frontal Eye Field^70,71^Superior Colliculus^72,73^and the Parietal Cortex^74,75^. While numerous studies have reported physiological correlates of vergence *eye-posture*, especially in the PPC^75–82^to our knowledge no study has conclusively demonstrated a causal role of a given area for vergence *eye-movements*. Overall, our understanding of the physiological underpinning of disjunctive eye-movements remains extremely sparse as compared to conjunctive eye-movements. However, in this study, we found that two effects known to modulate saccadic latencies and likely originating in the Superior Colliculus (SC), hemifield and size-latency effects, did not similarly modulate vergence latencies. This dissociation suggests that SC is not involved in the initiation of vergence eye-movements.

Functionally, the coordination between vergence and version eye-movements is interesting because multiple theories postulate use of an efference copy of eye-movements^83–88^. Almost all studies and theories focus exclusively on conjunctive eye-movements (but see^89^). The fact that vergence eye-movements are initiated independently from version eye-movements leads to interesting implications for these theories. For instance, the fact that vergence eye-movements are initiated earlier than saccades demonstrates that the visual system is able to select the disparity of a saccadic target and use this signal to drive vergence long before this signal reaches the fovea. Congruent with this idea, while our results show a lack of coordination between the *initiation* of saccadic and vergence eye-movements, it remains likely that the *selection* of these movements is coordinated^90^.

## Conclusion

In conclusion, we found that eccentricity and hemifield (lower vs. upper) modulated the initiation latency of saccadic eye-movements, but not the disparity of the targets. The opposite was true for vergence eye-movements: vergence initiation latencies varied with disparity sign and magnitude, but not the hemifield or eccentricity of the targets. Furthermore, there was a total lack of correlation between the initiation latency of saccades and vergence eye-movements. We also found that vergence latency distributions had a markedly reduced skewness, a hallmark of evidence accumulation, suggesting that vergence eye-movements are more reflexive, while saccadic eye-movements are more deliberative. Overall, all these elements clearly indicate that vergence and saccades initiation are functionally, and therefore probably also physiologically, completely dissociated.

## Methods

### Observers

Observers were 11 students and faculty at the Philipps Universitaet Marburg. All observers were naive to the purpose of the experiment except one author (BC). All observers had normal or corrected-to-normal vision and stereoacuity of 20 arcsec or better as measured with the Randot stereoacuity test (Precision Vision, La Salle, IL). Experiments were conducted in accordance with local and international regulations (Declaration of Helsinki). The protocol was approved by the institutional research ethics board of the Fachbereich Psychologie of the Philipps Universitaet Marburg (Aktenzeichen 2023-12k), and participants provided informed consent.

### Stimulus and procedure

Stimuli were presented on a VIEWPixx monitor (VPixx, Saint-Bruno, Canada) subtending 60 × 32 deg of visual angles at a viewing distance of 50 cm. Stimuli were presented dichoptically through red/green Wratten filters 25 and 58 (Kodak, Rochester, USA). Eye-movements were monitored binocularly at 1000 Hz using an Eyelink 1000 (SR-research, Ottawa, Canada). At the beginning of the experiment the eye-tracker was calibrated for each eye independently using a 9-dots calibration procedure.

The stimulus sequence is depicted in Fig. 1A. Each trial started with a 30 arcmin fixation mark displayed at the center of the monitor. After a pseudo-random delay (fixed 1000 ms + exponentially distributed random duration of mean 500 ms), the fixation mark was replaced by a high-contrast 12-deg diameter ring (20 arcmin thickness, ~ 50% contrast). The ring center was at ± 0, 2, 4 or 6 deg vertically relative to fixation and had a horizontal disparity of ± 0, 1 or 2 deg. The ring was displayed for a fixed-duration of 1500 ms, allowing observers to complete their eye-movement. Then, after a 500 ms blank period, a new fixation mark was displayed at the center of the monitor and the next trial was initiated as soon as both eyes were within 1 deg of the fixation mark. We collected 20 trials per condition, for a total of 680 trials.

### Estimation of eye-movement onsets

Eye traces were recoded without filtering (File Sample Filter in the Eyelink settings) and were smoothed offline with a 5 ms acausal Gaussian kernel for analyses. Because saccades were vertical and vergence eye-movements horizontal, we computed the mean version and vergence for each trial as follow:

$$\:S\left(t\right)=0.5\left({Y}_{L}\left(t\right)+{Y}_{R}\left(t\right)\right)\:\text{a}\text{n}\text{d}\:\text{G}\left(\text{t}\right)={X}_{L}\left(t\right)-{X}_{R}\left(t\right)$$

With S/G the version and vergence respectively, X/Y indicating the horizontal and vertical position of the eyes, and R/L denoting the Right and Left eyes.

On each trial, saccade onset was detected using a combined velocity and acceleration threshold: saccade onset was defined as the first time since target onset where both velocity was higher than 10 deg sec^−1^ and acceleration was higher than 250 deg sec^−2^. Adopting different methods for detecting saccadic latencies, such as different threshold values or a regression-based approach, led to similar conclusions.

A threshold-based approach for detecting vergence onset was unreliable due to the low velocities and accelerations of these types of eye-movements. Instead, we adopted a regression-based approach^24–26^. For each trial we estimated the first time at which vergence deviated from baseline by more than 30 arcmin. Then we fitted vergence traces with a piecewise linear function to the preceding 200 ms, to estimate the time at which vergence started deviating from baseline. Alternative techniques to measure vergence latency, such as thresholding position led to qualitatively similar, albeit more noisy results.

### Analyses

Since distributions of saccadic and vergence latencies exhibited a significant skewness, we computed the median of latencies for each observer and condition. A low fraction of trials (1.6% on average) contained express saccades, which we defined as saccadic latencies lower than 100 ms, and were removed from the analyses.

ANOVAs were performed in Matlab using the anovan function. Linear model fits were performed in Matlab using the fitglm function.

Skewness was defined as the third moment of the distributions:

$$\:{\stackrel{\sim}{\mu\:}}_{3}=\frac{E{(x-\mu\:)}^{3}}{{\sigma\:}^{3}}$$

With $\:\mu\:$ and $\:\sigma\:$ respectively the mean and standard deviation of the distribution. The expected skewness of an inverse-Gaussian distribution of identical mean and variance was estimated as^91^:

$$\:{\stackrel{\sim}{\mu\:}}_{3}=3\frac{\sigma\:}{\mu\:}$$

Saccadic peak velocities were computed as the maximum value (regardless of sign) within a time window of 0-150 ms after saccade onset. Vergence velocities were computed as the mean velocity within a time window of 50–150 ms after vergence onset. The choice of this window was meant to reflect the initial open-loop vergence response to disparity stimuli^45,92,93^. Choosing time windows of a different width or delay did not change the results qualitatively.

Saccadic spread was computed as the mean distance to the mean landing point, therefore analogous to a standard deviation:

$$\:D=\sum\:_{i}\frac{\sqrt{{\left({x}_{i}-\stackrel{-}{x}\right)}^{2}+{\left({y}_{i}-\stackrel{-}{y}\right)}^{2}}}{n}$$

With $\:D$ the spread, $\:{x}_{i}$ and $\:{y}_{i}$ the horizontal and vertical landing position for a given trial $\:i$, $\:\stackrel{-}{x}$ and $\:\stackrel{-}{y}$ their respective means and $\:n$ the number of trials.

Vergence error was computed as the Euclidian distance between the final vergence in a time window of 450–500 ms after vergence onset, and the vergence demand of the target (vergence demand of the fixation + disparity).

We computed version and vergence error over time as the Euclidian distance between eye-position and target center.

$$\:{E}_{S}\left(t\right)=abs\left(S\right(t)-{T}_{E})\:\text{a}\text{n}\text{d}\:{E}_{G}\left(t\right)=abs\left(G\right(t)-{T}_{D})$$

With $\:S$ and $\:G$ the eyes version and vergence as described previously, $\:{T}_{E}$ and $\:{T}_{D}$ the target elevation and disparity respectively. We then normalized these values by the maximum and minimum of the mean error ($\:\stackrel{-}{E}$) for a given observer and condition:

$$\:{E}^{*}\left(t\right)=\frac{E\left(t\right)-min\left(\stackrel{-}{E}\right)}{max\left(\stackrel{-}{E}\right)-min\left(\stackrel{-}{E}\right)}$$

## Electronic supplementary material

Below is the link to the electronic supplementary material.

Supplementary Material 1

Supplementary Material 2

## Data Availability

Data is provided in supplementary information files.
