# Supplementary material for: Dissociate triggering of conjunctive and disjunctive eye movements
Source: Sci Rep. 2025 Aug 11;15:29355. doi: 10.1038/s41598-025-12031-5 (PMC12339721; doi:10.1038/s41598-025-12031-5)
Supplement: Supplementary file 2 — Supplementary Material 2 [file 41598_2025_12031_MOESM2_ESM.pdf]

Supplementary Material

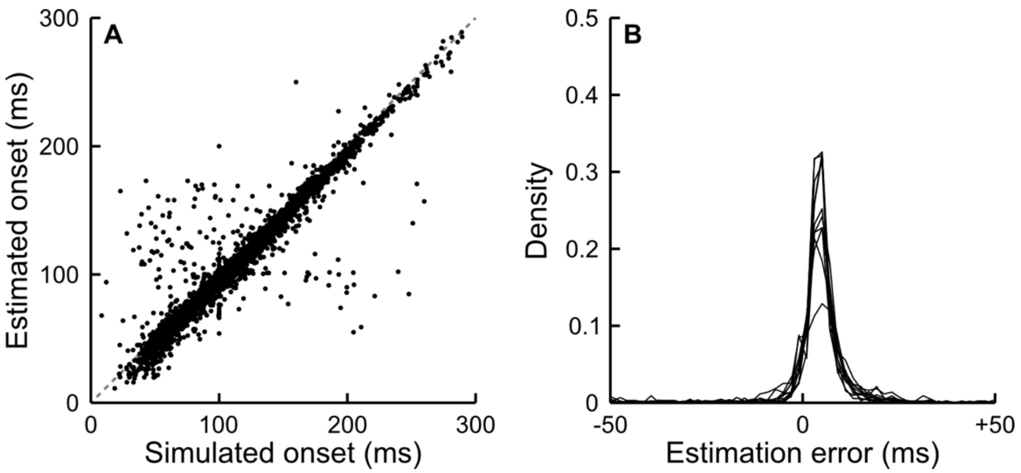

Figure S1. Estimation of the regression-based vergence onset detection algorithm. (A) Estimated onset (ordinates) as a function of simulated onset (abscissa) for all trials and observers. (B) Distribution of vergence onset estimation error for each observer separately (lines).

To evaluate the accuracy of our regression-based estimation of vergence latencies, we simulated ideal vergence eye-movements (rectified exponential ramps): for each trial, we generated an exponential ramp of identical latency, initial velocity and final vergence. We then corrupted this ideal eye-movement by noise of equal magnitude as the noise in a 50 ms window prior target onset. We then estimated the onset of these simulated eye-traces using an identical algorithm as for the real eye-traces. Figure S1A plots estimated onsets as a function of simulated onsets for all trials and observers (6020 trials). Except for a negligible fraction of trials, all estimated onsets are tightly grouped around the unity line. The median correlation across observers between the vergence onset estimated from the real data and simulated data was 0.92. The median latency error across observers was 4.5 ms, indicating that this approach tends to slightly underestimate the true onset of the vergence eye-movement. The median standard deviation of the latency errors across observers between simulated and estimated latencies was 4.3 ms. This corresponds to a median fraction of variance within condition of 1.1%, which is unlikely to affect the overall conclusions of our analyses.

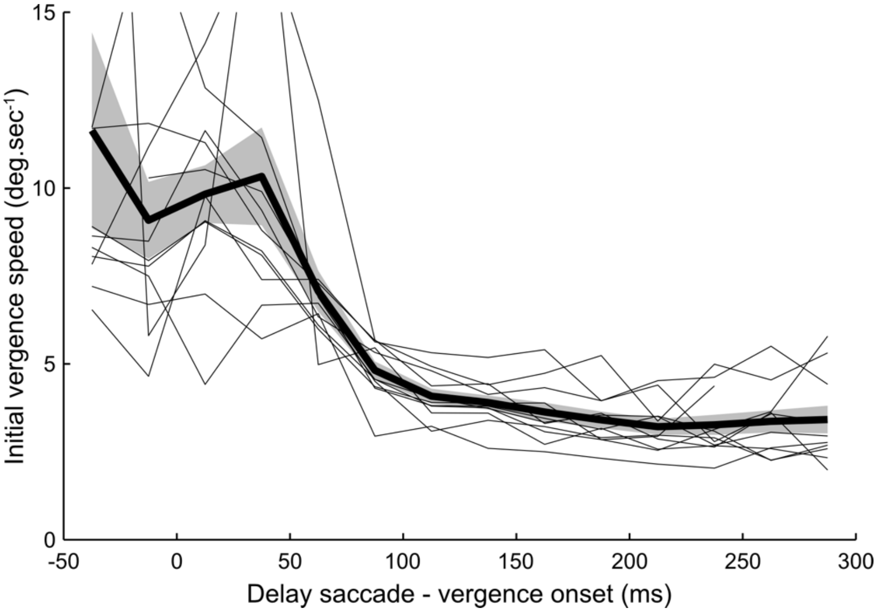

Figure S2. Saccadic enhancement of vergence speed: Mean absolute initial vergence speed (ordinates) for each observer (thin lines), mean across observers (thick line) and standard error (shaded area); as a function of the delay between saccade onset and vergence onset (abscissa). Here positive delays mean that the saccade onset occurs after vergence onset.

Vergence speed tends to be enhanced around the onset of saccades. Busettini & Mays (2005)[55] reported that this effect depends systematically on the relative onset of saccadic and vergence eye-movements, with strongest enhancements when both eye-movements are initiated at the same time. Figure S2 plots absolute initial vergence speed (mean vergence speed within a window 50 to 100 ms after vergence onset) as a function of the delay between the initiation of the vergence eye-movement and the saccadic eye-movement. A positive delay means that the saccade was initiated after vergence. As Busettini & Mays (2005), we find that vergence speed is strongly enhanced for small delays, and decreases for larger delays. This effect is significant for all observers (distribution of regression slopes on resampled datasets for trials with delays between 0 and 300ms, $p<0.001$, uncorrected for multiple comparisons).

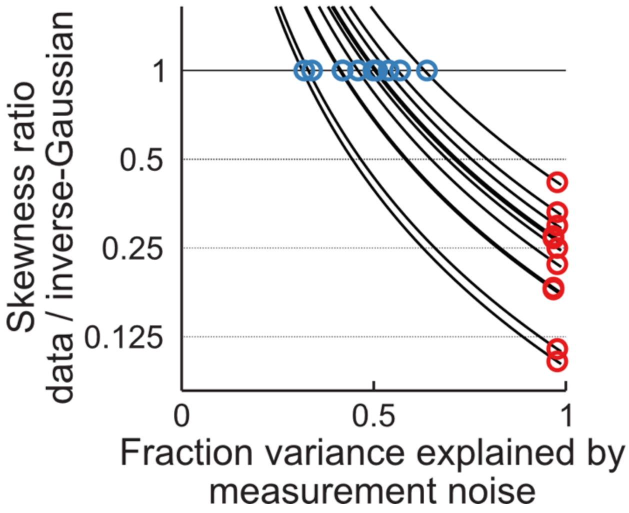

Figure S3. Effect of measurement noise on skewness: Skewness ratio of an inferred distribution of vergence onsets (ordinates, same as Figure 3D), that has been corrupted by varying amounts of measurement noise (abscissa) for each observer separately (black lines). A fraction of 0 means that all variance in the distribution is measurement noise, and a faction of 1 none of the variance. Blue dots indicate the amount of measurement noise that would be required to have observed the measured skewness while the true skewness of the distribution was identical to that of an inverse-Gaussian. Red dots indicate our best estimation of measurement noise caused by the regression-based approach (from Figure S1).

An overestimation of a distribution variance leads to an underestimation of this distribution skewness. Consequently, the reduced skewness of the vergence onset distributions could be a simple artifact of a higher measurement noise. To assess this possible confound, we computed the same log-skewness-ratio as in Figure 3D assuming that part of the variance is caused by measurement noise. A value of 0 means that all of the variance in the distribution is caused by measurement noise, and a value of 1 none of this variance. A skewness ratio of 1 means that the distribution true skewness is equal to that of an inverse-Gaussian. The blue circles indicate the amount of measurement noise that would be required to have observed the vergence onsets skewness we measured, while the true skewness of the distribution was identical to that of an inverse-Gaussian. The median fraction across observers is 0.51 (thus corresponding to 49% of the variance caused by measurement noise). The red circles indicate our best estimate of measurement noise from Figure S1. Overall, this analysis indicates that the skewness of the vergence latency distributions is truly lower than that of an inverse-Gaussian.

Table S1: 4-way ANOVA on saccadic latencies.

Source	Sum	Sq.	d.f.	Mean	Sq.	F	Prob>F
hemifield	26	235	1	26.23	55	74.5	0
eccentricity	45	803	2	22.90	14	65.03	0
disparity sign	1	129	1	1.12	87	3.21	0.0747
disparity value	0	13	1	0.01	34	0.04	0.8458
hemifield:eccentricity	0	79	2	0.03	97	0.11	0.8934
hemifield:disparity sign	0	1	1	0.00	1	0	0.9572
hemifield:disparity value	0	36	1	0.36		1.02	0.313
eccentricity:disparity sign	0	551	2	0.27	53	0.78	0.4588
eccentricity:disparity value	0	46	2	0.02	32	0.07	0.9364
disparity sign:disparity value	0	2	1	0.00	2	0.01	0.9393
hemifield:eccentricity:disparity sign	0	3	2	0.01	52	0.04	0.9579
hemifield:eccentricity:disparity value	0	15	2	0.00	73	0.02	0.9795
hemifield:disparity sign:disparity value	0	23	1	0.02	25	0.06	0.8005
eccentricity:disparity sign:disparity value	0	38	2	0.01	9	0.05	0.9476
hemifield:eccentricity:disparity sign:disparity value	0	13	2	0.06	52	0.19	0.8311
Error	84	521	240	0.35	22		
Total	158	977	263				

Table S2: 4-way ANOVA on vergence latencies.

Source	Sum	Sq.	d.f.	Mean	Sq.	F	Prob>F
hemifield	0	19	1	0.0	19	0.01	0.9147
eccentricity	1	344	2	0.6	72	0.41	0.6626
disparity sign	92	961	1	92.9	61	57.05	0
disparity value	147	181	1	147.1	81	90.32	0
hemifield:eccentricity	0	233	2	0.1	16	0.07	0.9311
hemifield:disparity sign	1	246	1	1.2	46	0.76	0.3828
hemifield:disparity value	2	319	1	2.3	19	1.42	0.2341
eccentricity:disparity sign	0	993	2	0.4	96	0.3	0.7378
eccentricity:disparity value	1	28	2	0.5	14	0.32	0.7297
disparity sign:disparity value	1	728	1	1.7	28	1.06	0.3042
hemifield:eccentricity:disparity sign	6	72	2	3.0	36	1.86	0.1574
hemifield:eccentricity:disparity value	0	914	2	0.4	57	0.28	0.7558
hemifield:disparity sign:disparity value	2	471	1	2.4	71	1.52	0.2194
eccentricity:disparity sign:disparity value	1	32	2	0.6	6	0.4	0.6674
hemifield:eccentricity:disparity sign:disparity value	2	875	2	1.4	37	0.88	0.4153
Error	391	104	240	1.6	3		
Total	653	805	263				

Table S3: 4-way ANOVA on saccadic spread.

Source	Sum	Sq.	d.f.	Mean	Sq.	F	Prob>F
hemifield	2	443	1	2.4	43	2.03	0.1556
eccentricity	341	478	2	170.7	39	141.79	0
disparity sign	0	3	1	0.0	3	0	0.9591
disparity value	0	782	1	0.7	82	0.65	0.4212
hemifield:eccentricity	0	632	2	0.3	16	0.26	0.7695
hemifield:disparity sign	0	5	1	0.0	5	0	0.9477
hemifield:disparity value	0	115	1	0.1	15	0.1	0.7575
eccentricity:disparity sign	1	418	2	0.7	9	0.59	0.5559
eccentricity:disparity value	0	753	2	0.3	76	0.31	0.7318
disparity sign:disparity value	0		1	0		0	0.9996
hemifield:eccentricity:disparity sign	0	13	2	0.0	65	0.05	0.9477
hemifield:eccentricity:disparity value	0	858	2	0.4	29	0.36	0.7008
hemifield:disparity sign:disparity value	0	1	1	0.0	1	0	0.9759
eccentricity:disparity sign:disparity value	0	3	2	0.0	1	0	0.9989
hemifield:eccentricity:disparity sign:disparity value	0	135	2	0.0	68	0.06	0.9454
Error	289	7	240	1.2	4		
Total	637	762	263				

Table S4: 4-way ANOVA on vergence endpoints.

Source	Sum	Sq.	d.f.	Mean	Sq.	F	Prob>F
hemifield	0.0	437	1	0.043	69	0.3	0.5851
eccentricity	1.2	64	2	0.631	98	4.32	0.0143
disparity sign	0.1	751	1	0.175	14	1.2	0.2749
disparity value	1.2	25	1	1.225	1	8.38	0.0041
hemifield:eccentricity	0.1	29	2	0.064	52	0.44	0.6437
hemifield:disparity sign	0.0	45	1	0.045	3	0.31	0.5794
hemifield:disparity value	0.0	102	1	0.010	18	0.07	0.7921
eccentricity:disparity sign	0.1	799	2	0.089	97	0.62	0.5413
eccentricity:disparity value	0.0	128	2	0.006	42	0.04	0.957
disparity sign:disparity value	0.0	79	1	0.007	93	0.05	0.8161
hemifield:eccentricity:disparity sign	0.0	419	2	0.020	93	0.14	0.8667
hemifield:eccentricity:disparity value	0.0	135	2	0.006	75	0.05	0.9549
hemifield:disparity sign:disparity value	0.0	302	1	0.030	15	0.21	0.6502
eccentricity:disparity sign:disparity value	0.1	39	2	0.069	52	0.48	0.6222
hemifield:eccentricity:disparity sign:disparity value	0.0	294	2	0.014	7	0.1	0.9044
Error	35.0	915	240	0.146	21		
Total	38.4	382	263				
